# Compound Solution of Iodine in Chronic Diarrhoea

**Published:** 1871-03

**Authors:** E. L. Shurly

**Affiliations:** Manistee, Mich.


ART. V.—*Compound Solution of Iodine in Chronic Diarrhoea* By E. L. SHURLY, Manistee, Mich.

Of all diseases abounding in this region, we have found none more intractable or formidable than chronic diarrhoea, which is always of malarial origin, being preceded or accompanied by intermittent or remittent fever, and more or less hepatic or splenic enlargement; while the patient's history and appearance betokened the amount of illness he must have undergone before the onset of the diarrhoea.

For the treatment of these cases, heretofore, we have persistently employed all of the astringents, tonics, &c., of reputed efficiency, alone, and in connection with quinia! Only succeeding, however, in limiting the disease until "Jack Frost" had made his welcome. appearance, when the patient would almost or wholly recover. Driven by these unsatisfactory results to seek some new mode of action, we accordingly, in the next case which presented itself, prescribed five drops of the com. sol. iodine, (Lugol's solution,) in one-half tumbler of water, four times daily, with a view of reducing the hepatic enlargement present, on the theory of causation; when, after three days of treatment, the fact was revealed that the iodine had entirely controlled the diarrhoea, and greatly relieved the patient.

Thus several more cases of this class were treated with equally satisfactory results—some having hepatic or splenic enlargement, while some were free from these complications; all of the cases, however, being associated with miasmatic poisoning.

We have, therefore, been induced to publish this note, in order that the virtue of this remedy, in respect to curing chronic diarrhoea in different localities, and under different circumstances, may be tested and made known.

